# Supplementary material for: Symmetry breakdown of 4,4″-diamino-p-terphenyl on a Cu(111) surface by lattice mismatch
Source: Nat Commun. 2018 Aug 16;9:3277. doi: 10.1038/s41467-018-05719-y (PMC6095862; doi:10.1038/s41467-018-05719-y)
Supplement: Supplementary file 1 — Supplementary Information [file 41467_2018_5719_MOESM1_ESM.pdf]

Supporting Information

Symmetry breakdown of 4,4''-Diamino-*p*-terphenyl on a Cu(111) surface by lattice mismatch

Qigang Zhong[1], Daniel Ebeling[2], Jalmar Tschakert[2], Yixuan Gao[3], Deliang Bao[3], Shixuan Du[3]*, Chen Li[4], Lifeng Chi[1]*, André Schirmeisen[2]*

(1) Institute of Functional Nano & Soft Materials (FUNSOM), Jiangsu Key Laboratory for Carbon-Based Functional Materials and Devices, Soochow University, Suzhou 215123, P. R. China

(2) Institute of Applied Physics, Justus-Liebig University, Heinrich-Buff-Ring 16, 35392 Giessen, Germany

(3) Institute of Physics & University of Chinese Academy of Sciences, Chinese Academy of Sciences, Beijing 100190, P. R. China

(4) School of Environment and Civil Engineering, Dongguan University of Technology, Dongguan 523808, P. R. China

*email: daniel.ebeling@ap.physik.uni-giessen.de; sxdu@iphy.ac.cn; chilf@suda.edu.cn

Table of content

Supplementary Figure 1. Reversible switching between adsorption types I and II. 3

Supplementary Figure 2. 360° STM manipulation of a single DATP molecule on Cu(111). 4

Supplementary Note 1. Manipulation procedure used in Supplementary Figure 2. 5

Supplementary Figure 3. Statistics on the jumping rate of DATP Type I. 6

Supplementary Figure 4. Energy barriers measured with a lower bias (30 mV). 7

Supplementary Figure 5. Tip-height dependence of the jumping rate. 8

Supplementary Figure 6. Lateral adsorption geometries of DATP Type I. 9

Supplementary Figure 7. 1D and 2D frequency shift spectroscopy over a DATP Type I molecule adsorbed on Cu(111). 10

Supplementary Figure 8. Simulated adsorption structures of DATP on Cu(111). 11

Supplementary Figure 9. Adsorption geometries of DATP on Au(111). 12

Supplementary Figure 10. STM overview of DATP-TPCA assembly on Cu(111). 13

Supplementary Figure 11. Hydrogen bonding between DATP and TPCA molecules. 14

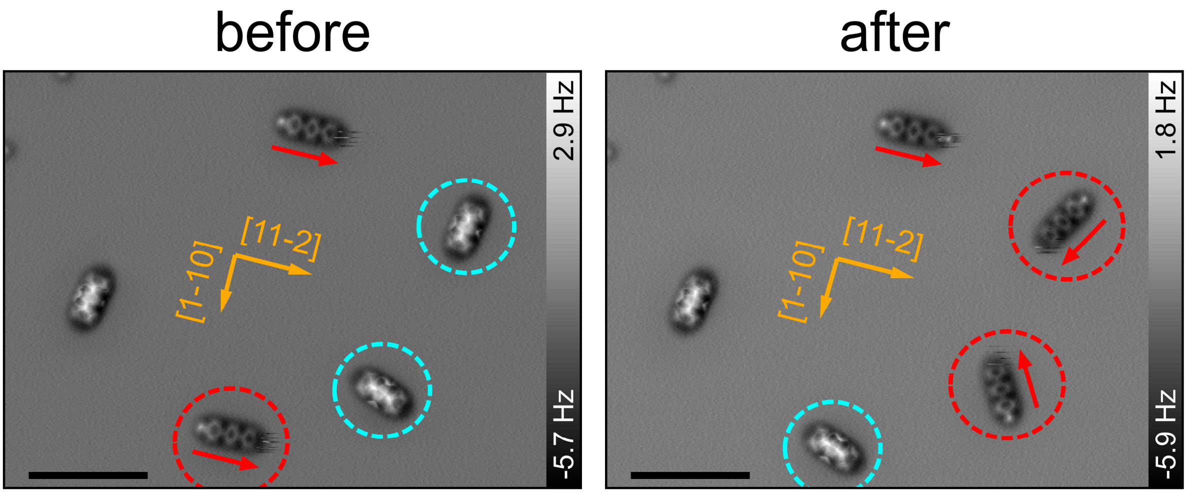

Supplementary Figure 1. Reversible switching between adsorption types I and II. (**left**) AFM overview image with two type I and three type II DATP molecules. (**right**) AFM overview of the same area after tip manipulation showing two transitions from type II to type I (blue circles in left scan) and one transition from type I to type II (red circle in left scan). Parameters: the tip-heights Δz are -90 pm, relative to a STM set point of 100 mV, 10 pA on bare Cu surfaces. Scale bars: 3 nm.

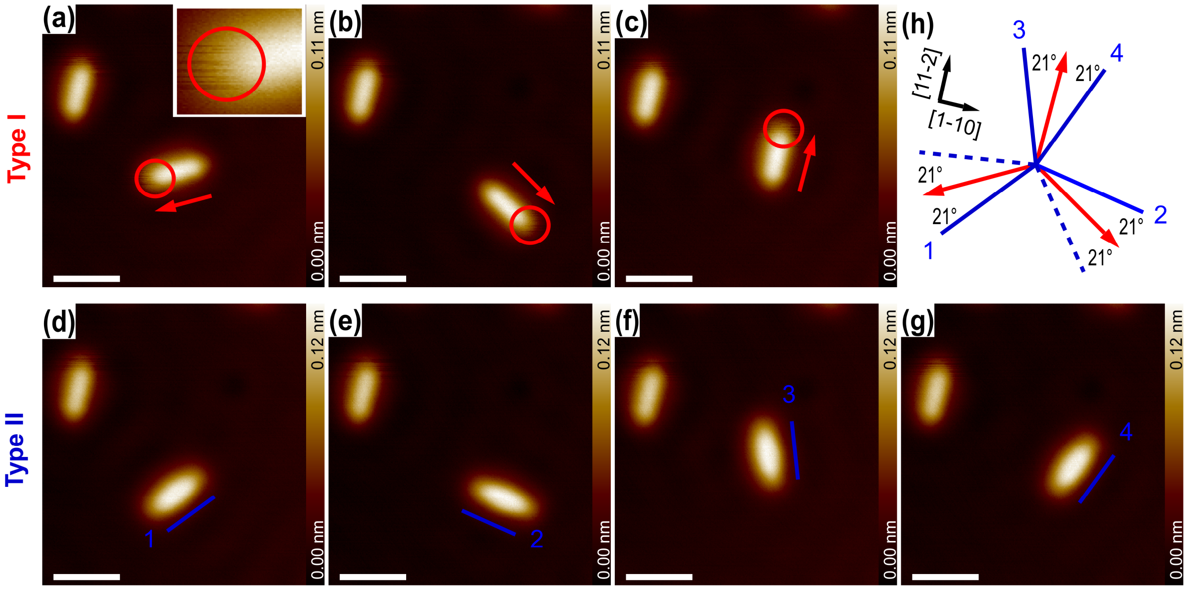

Supplementary Figure 2. 360° STM manipulation of a single DATP molecule on Cu(111). (a-c) STM images of type I adsorption structure. Type I exists in three orientations with an interval of 120° from one to another (three-fold symmetry). The fuzzy ends were marked with red circles. A zoom-in image of the fuzzy end was inserted in (**a**). (**d-g**) STM images of Type II. Type II structures were found to be rotated by 21° with regard to type I structures. (**h**) Diagram of the orientations of DATP on Cu(111). The red arrows represent the three possible orientations of Type I structures (arrow tips indicate fuzzy ends). Type I structures align with the crystallographic [11-2] direction. The blue lines refer to the orientations of Type II, where the four solid blue lines correspond to images (**d-g**) and the dashed blue lines stand for another two possible orientations of Type II. Parameters: STM set point for imaging: 100 mV, 10 pA; for manipulation: 2 mV, 100 pA, voltage is applied to the sample while the tip is grounded. Scale bars: 2 nm.

Supplementary Note 1. Manipulation procedure used in Supplementary Figure 2. Manipulation has been performed in the STM mode (with a DATP tip) with certain imaging and manipulation parameters (see caption of Supplementary Fig. 2). Since the transition events involve a rotation of the molecule, the switching could be triggered by i) pausing the scan and placing the tip next to one corner of the molecule (i.e., next to one of the amine groups), ii) setting appropriate manipulation parameters (certain tunneling set point and gap voltage), and iii) moving the tip perpendicularly to the longitudinal axis of the molecule towards its corner in order to rotate it. After each manipulation procedure a snapshot of the new orientation of the molecule has been taken. With proper imaging and manipulation parameters the described procedure offers a success rate on the order of 90 %. Rarely, we also observed no rotation of the molecule or even a backward jump into the previous (more stable) adsorption state.

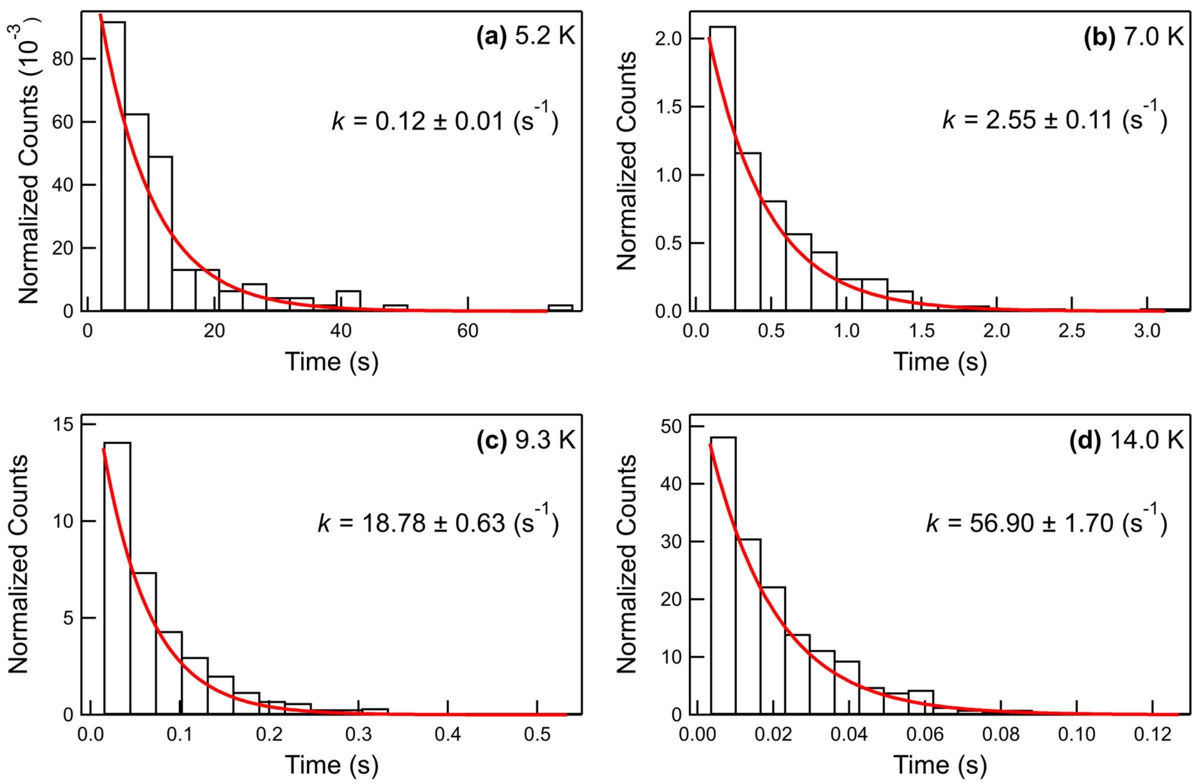

Supplementary Figure 3. Statistics on the jumping rate of DATP Type I. (**a-d**) Histogram of the jumping periods (for total jumps) of DATP Type I at four different temperatures: 5.2 K, 7.0 K, 9.3 K, 14.0 K. Note that the jumping rate is normalized. The histograms are fitted with a simple exponential function ($y = ke^{-kx}$) to acquire the corresponding jumping rate k. In total, 118, 272, 1876 and 639 jumps are counted at 5.2 K, 7.0 K, 9.3 K, 14.0 K, respectively. The errors given for k correspond to +/- one standard deviation.

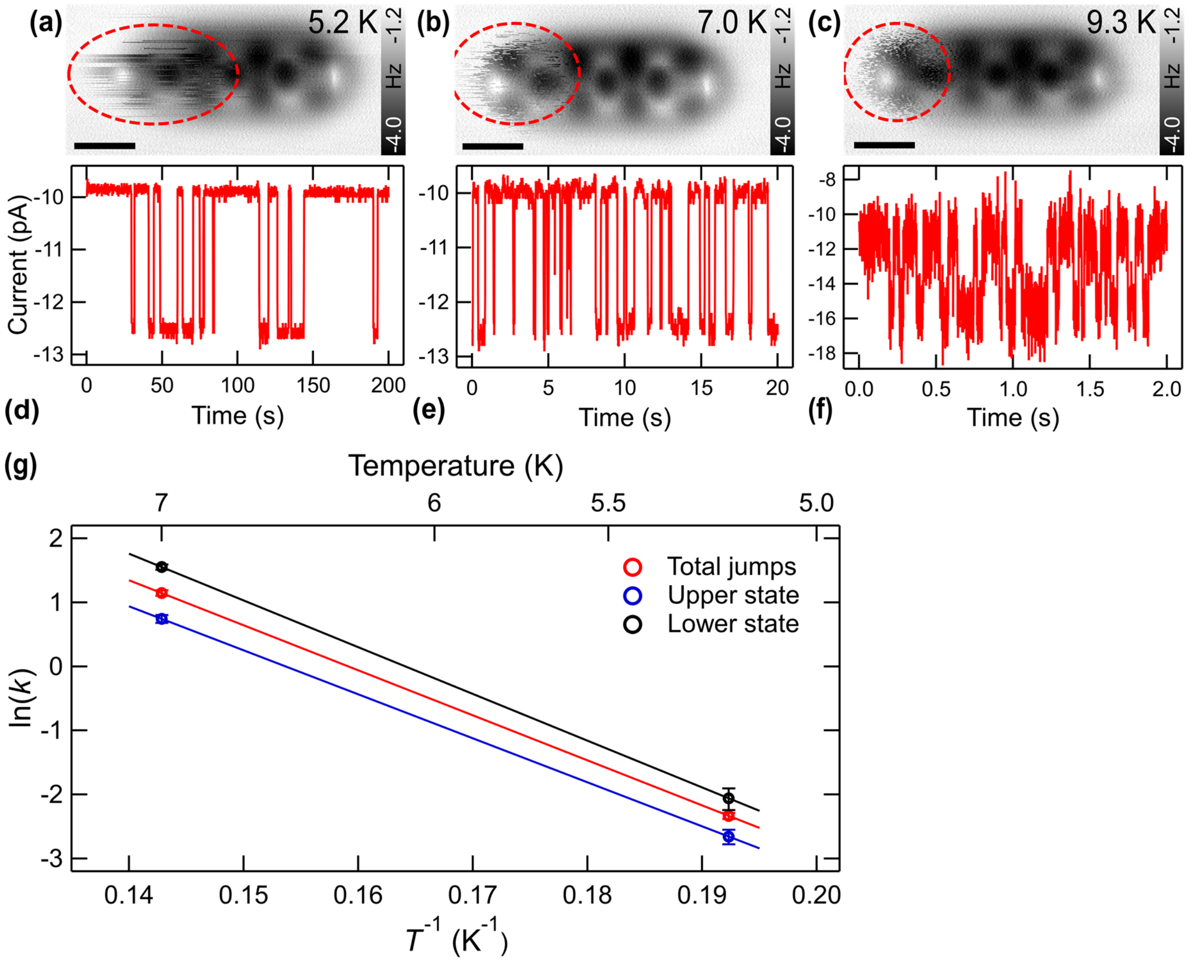

Supplementary Figure 4. Energy barriers measured with a lower bias (30 mV). (**a-c**) AFM images of a Type I DATP molecule taken at 5.2 K, 7.0 K and 9.3 K, respectively. (The tip-heights Δz are (**a**) -90 pm, (**b**) -85 pm, (**c**) -100 pm, relative to a STM set point of 100 mV, 10 pA on bare Cu surfaces.) (**d-f**) Corresponding current vs. time traces $I(t)$. (Parameters used for measuring the telegraph signal: V_{bias} = 30 mV, tip height Δz = -10 pm with respect to the STM tunneling set point of 100 mV, 10 pA on the bare Cu(111) surface.) (**g**) A graph of the natural logarithm of the jumping rate ln(k) *vs.* 1/T for the total jumps (red circles), jumps into the upper state (blue circles) and jumps in the lower state (black circles). An energy barrier (E_α) of 6.07 ± 0.13 meV and a pre-exponential factor (A) of $e^{11.20 \pm 0.31}$ s^{-1} for the total jumps are given by fitting the transformed Arrhenius' equation ($\ln k = (-E_\alpha/k_B)(1/T) + \ln A$, where k_B is the Boltzmann constant) to the two points of 5.2 K and 7.0 K. (For the upper state: E_α = 5.93 ± 0.30 meV, $A = e^{(10.58 \pm 0.56)}$ s^{-1}; For the lower state: E_α = 6.32 ± 0.37 meV, $A = e^{(12.03 \pm 0.65)}$ s^{-1}.) The vertical error bars in (**g**) are derived from the standard deviation of k. Scale bars: 0.5 nm.

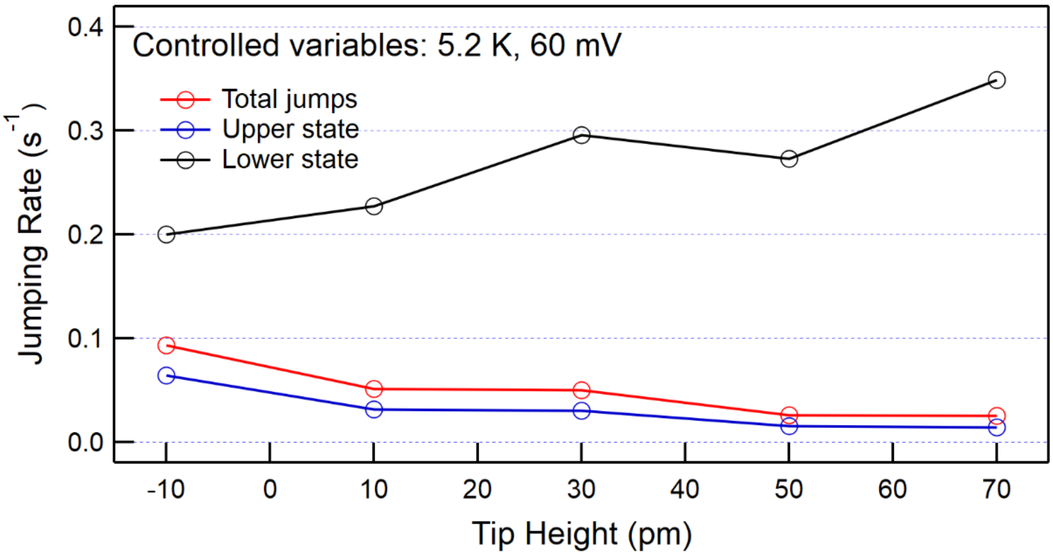

Supplementary Figure 5. Tip-height dependence of the jumping rate. Jumping rate vs. tip-height line chart. The total jumping rate (red circles) decays with the increase of the tip-height, which indicates that the tip induces the hopping of DATP type I. The jumping rate of the upper state (blue circles) decreases while the jumping rate of the lower state (black circles) increases with the rise of the tip, which suggests that the upper state is favorable while the lower state is unfavorable. The temperature (5.2 K) and the bias (60 mV) are control variables in this case.

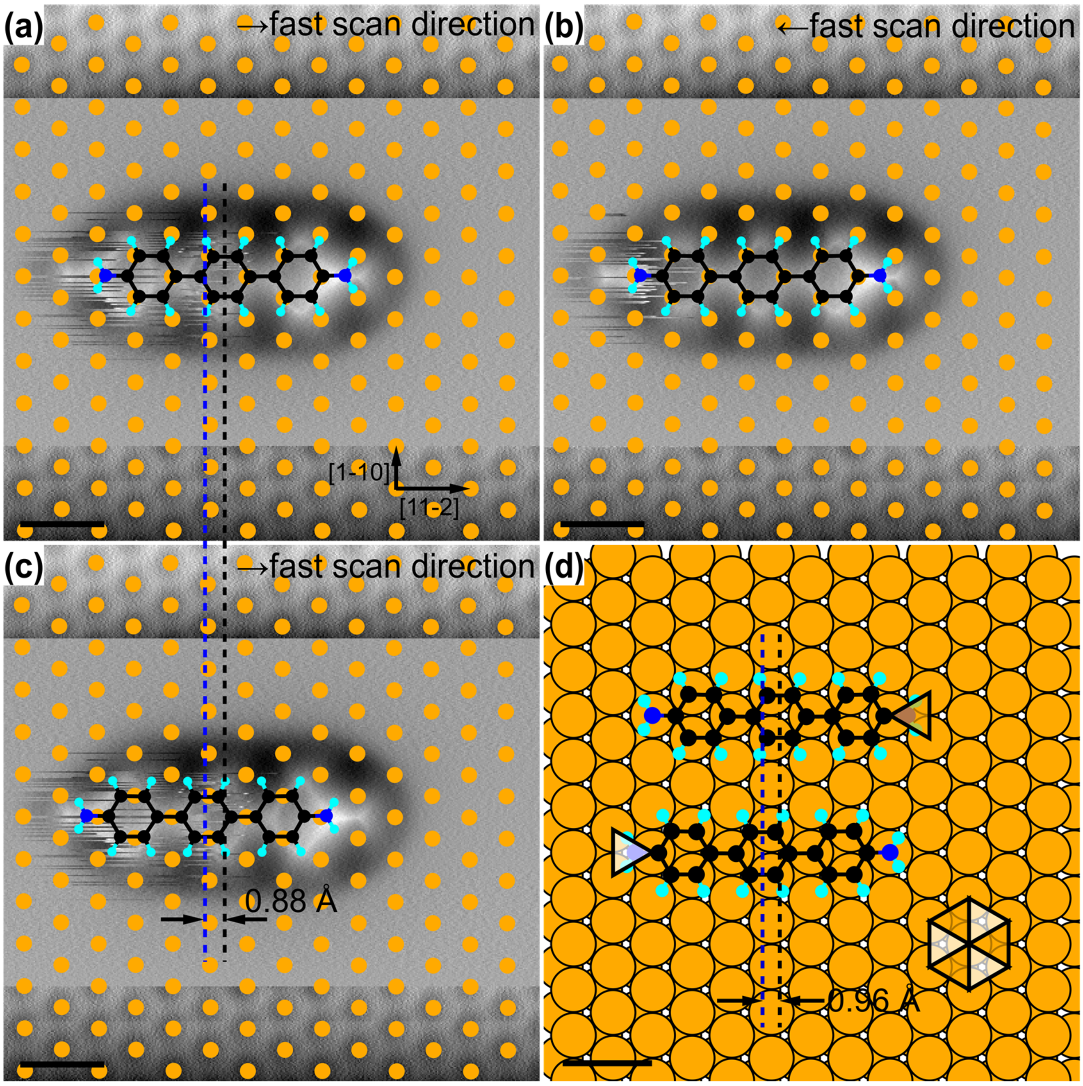

Supplementary Figure 6. Lateral adsorption geometries of DATP Type I. (**a-c**) AFM images of a DATP molecule on Cu(111) with atomic resolution of the Cu(111) surface layer at the top and the bottom parts of the images. Yellow dots indicate Cu(111) top sites. Δ*z* = -370 pm for atomic resolution of the Cu surface, Δ*z* = -110 pm for imaging the molecule. (**a**,**c**) and (**b**) have the opposite fast scan directions. In (**a**,**c**) two different adsorption positions with a shift of 0.88 Å in [11-2] direction were imaged. Accordingly, two same DATP molecular models were fitted to the two positions (cf. type IA and type IB in manuscript Fig. 3). In (**b**) only one adsorption position was observed which was fitted to using the same molecular model. (**d**) Anticipated model of the two different adsorption positions. The *hcp* and *fcc* hollow sites of Cu(111) where one end group of each molecule was located were marked with yellow and white filled triangles separately. Please note that the molecular structures, which are fitted to the images are in good agreement with the computed structures for DATP type IA and type IB molecules (see Fig. 3c,d). Scale bars: 0.5 nm.

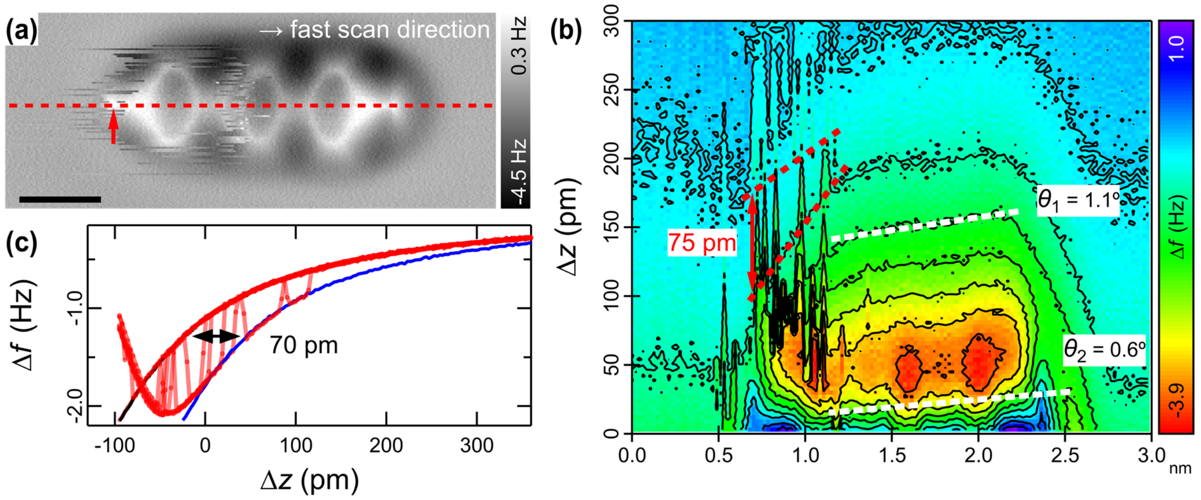

Supplementary Figure 7. 1D and 2D frequency shift spectroscopy over a DATP Type I molecule adsorbed on Cu(111). (**a**) AFM image of a Type I DATP molecule. The fast scan direction is from left to right. Tip-height $\Delta z = 0$ pm, relative to a STM set point of 100 mV, 10 pA on bare Cu surfaces. Scanning speed: 7.2 s/line. (**b**) 2D frequency shift vs z map along the red dashed line in (**a**) containing 150 single spectroscopy curves. White dashed lines reveal tilting of non-fuzzy part of the molecule. Jumps in z-direction at fuzzy part of molecule are indicated by red dashed lines. Duration for 2D map: 12.5 min. The Δz axis in the 2D frequency shift map is given with regard to the tip height in image (**a**). (**c**) Eight frequency shift vs z curves (red lines) taken above the fuzzy end of the molecule (position marked in (**a**) with a red arrow) are overlaid in one graph. Please note that these force curves are done with a different CO tip than that used for the 2D frequency shift map. Jumps between two states are observed (indicated by black and blue curves, the blue curve is shifted by 70 pm in z-direction with regards to the black one). Scale bar: 0.5 nm.

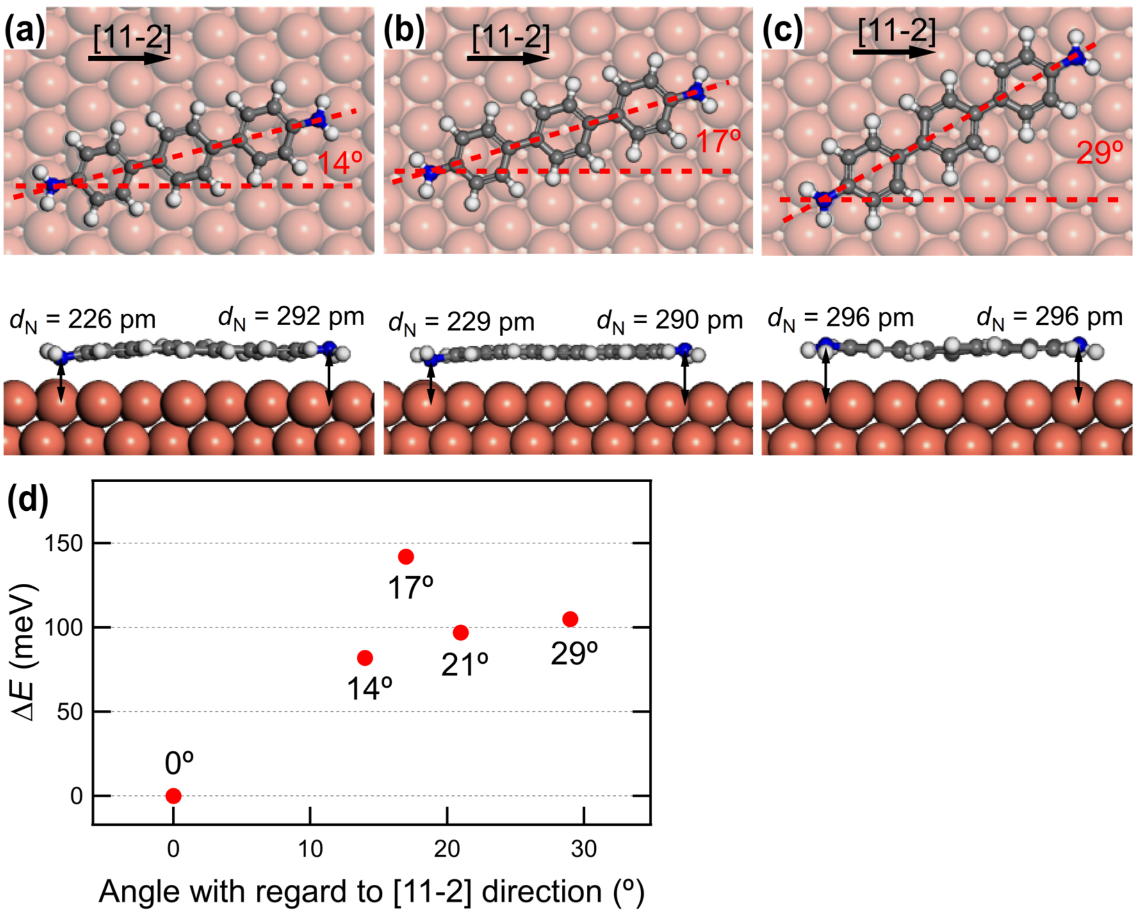

Supplementary Figure 8. Simulated adsorption structures of DATP on Cu(111). (**a-c**) Three more DFT calculated adsorption structures of DATP on Cu(111). The longitudinal axis of the DATP molecule is rotated by 14°, 17° and 29° with regard to the [11-2] direction for the three structures respectively. (**d**) Energy (ΔE) vs adsorption angle graph showing the energy differences of all the calculated structures relative to the Type I structure (0°).

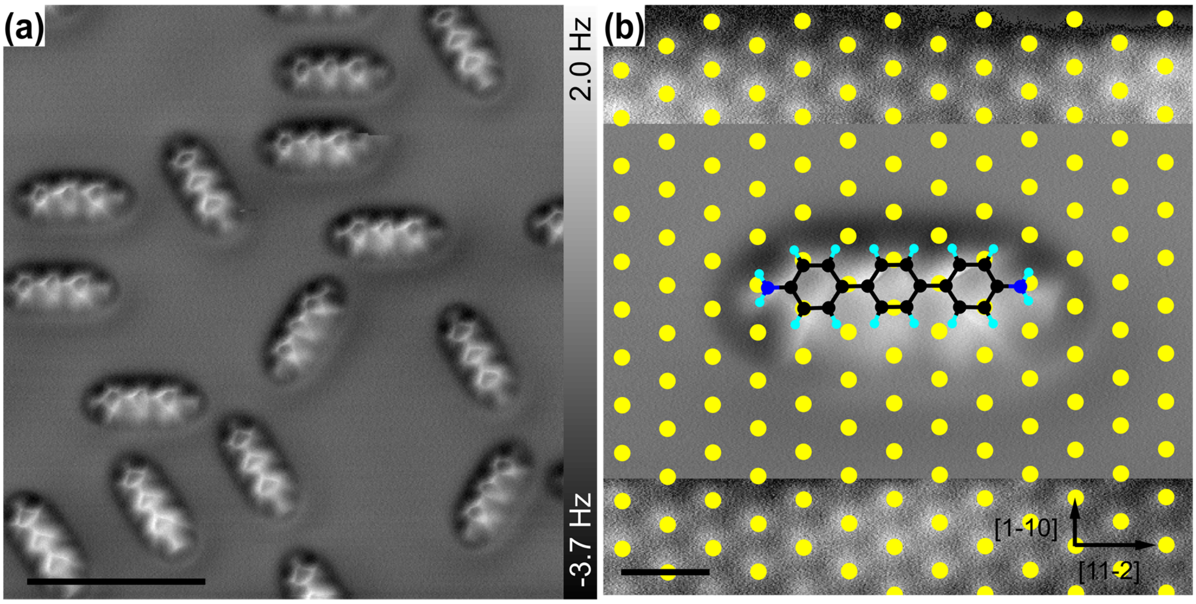

Supplementary Figure 9. Adsorption geometries of DATP on Au(111). (**a**) AFM overview of DATP molecules adsorbed on Au(111). (Δz = 0 pm) (**b**) AFM image of a single DATP molecule with atomic resolution of the Au(111) surface at the top and bottom parts of the image. (Δz = -300 pm for atomic resolution of the Au surface, Δz = -45 pm for imaging the molecule.) Bright yellow dots indicate Au(111) top sites. A DATP molecular model was fitted to the imaged DATP structure. The position of the fitted structure is in remarkable agreement with the computed structure. (see Fig. 4d in manuscript) (Tip heights (Δz) are set with respect to a tunneling set point of 100 mV, 10 pA on bare Au(111) surfaces.) Scale bars: (**a**) 3 nm, (**b**) 0.5 nm.

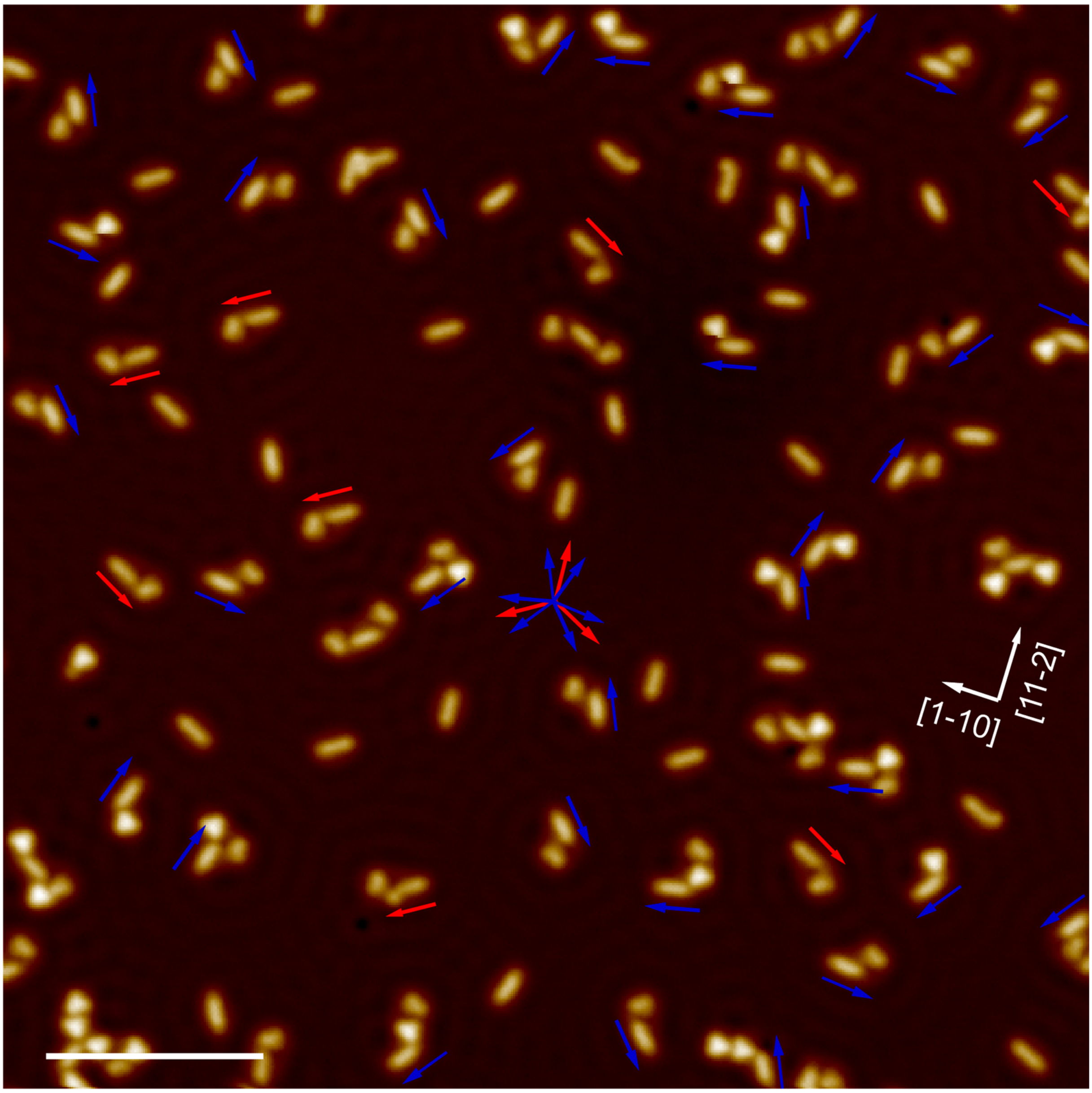

Supplementary Figure 10. STM overview of DATP-TPCA assembly on Cu(111). The image is acquired after cold evaporation (T_{sam} < 100 K). All the single-end occupied DATP molecules are marked with arrows, where red arrows and blue arrows represent DATP Type I and Type II, respectively. In total 12 of these overview scans have been performed at different sample positions and were used for the statistical analysis. Scale bar: 10 nm.

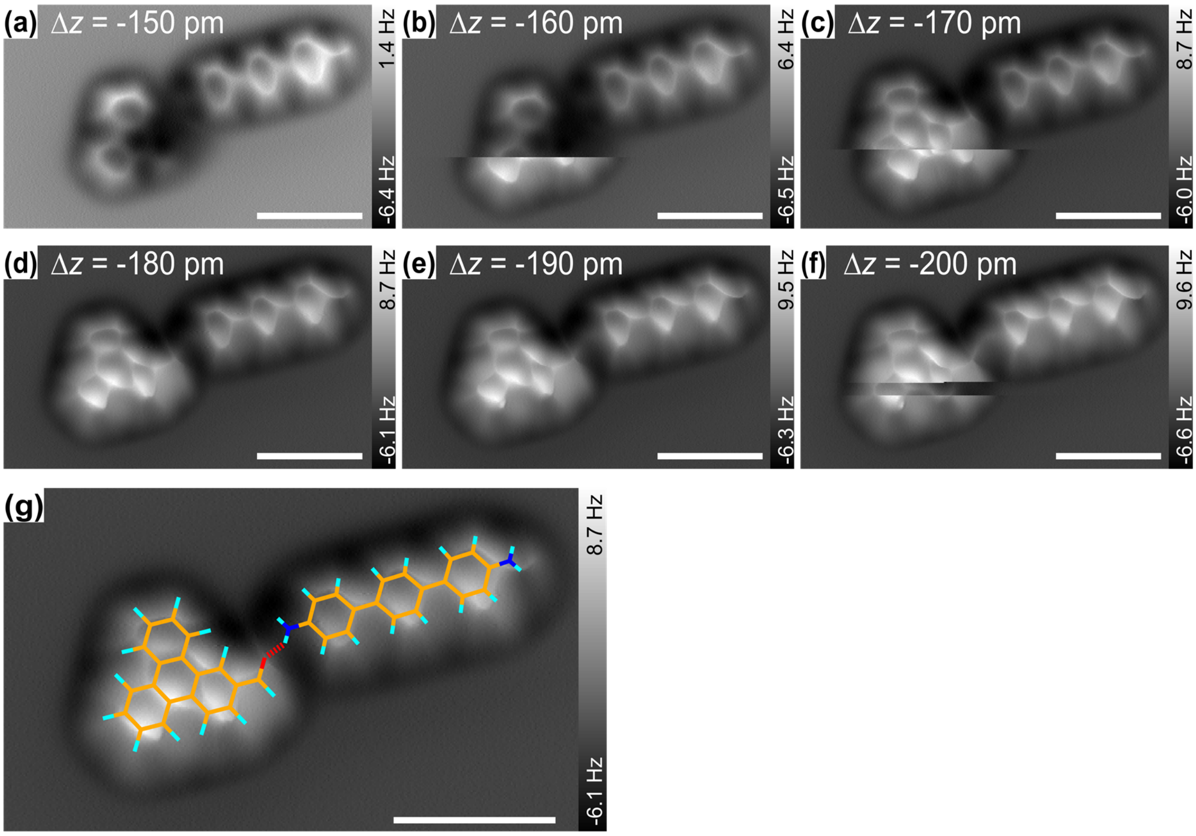

Supplementary Figure 11. Hydrogen bonding between DATP and TPCA molecules. (**a-f**) Series of constant height AFM images of DATP-TPCA cluster (see Fig. 5b in manuscript). The series of scans reveals orientational changes of the TPCA molecule while the DATP does not change its orientation. Hence, the two molecules are not covalently coupled. (**g**) Same scan as in (**d**) with overlaid molecular models. The molecules are connected via an $O \cdots H - N$ hydrogen bond (see red dashed line). Scale bars: 1 nm.
